# The ratio of hemoglobin to red cell distribution width as a novel prognostic parameter in esophageal squamous cell carcinoma: a retrospective study from southern China

**DOI:** 10.18632/oncotarget.9516

**Published:** 2016-05-20

**Authors:** Peng Sun, Fei Zhang, Cui Chen, Xiwen Bi, Hang Yang, Xin An, Fenghua Wang, Wenqi Jiang

**Affiliations:** ^1^ State Key Laboratory of Oncology in South China, Collaborative Innovation Center for Cancer Medicine, Guangzhou, Guangdong Province 510060, P. R. China; ^2^ Department of Medical Oncology, Sun Yat-Sen University Cancer Center, Guangzhou, Guangdong Province 510060, P. R. China; ^3^ Department of Oncology, The First Affiliated Hospital, Sun Yat-Sen University, Guangzhou, Guangdong Province 510080, P. R. China

**Keywords:** esophageal squamous cell carcinoma (ESCC), hemoglobin, red cell distribution width (RDW), prognosis, survival

## Abstract

**Background:**

We propose a novel prognostic parameter for esophageal squamous cell carcinoma (ESCC)—hemoglobin/red cell distribution width (HB/RDW) ratio. Its clinical prognostic value and relationship with other clinicopathological characteristics were investigated in ESCC patients.

**Results:**

The optimal cut-off value was 0.989 for the HB/RDW ratio. The HB/RDW ratio (*P*= 0.035), tumor depth (*P* = 0.020) and lymph node status (*P*<0.001) were identified to be an independent prognostic factors of OS by multivariate analysis, which was validated by bootstrap resampling. Patients with a low HB/RDW ratio had a 1.416 times greater risk of dying during follow-up compared with those with a high HB/RDW (95% CI = 1.024–1.958, *P* = 0.035).

**Materials and Methods:**

We retrospectively analyzed 362 patients who underwent curative treatment at a single institution between January 2007 and December 2008. The chi-square test was used to evaluate relationships between the HB/RDW ratio and other clinicopathological variables; the Kaplan–Meier method was used to analyze the 5-year overall survival (OS); and the Cox proportional hazards models were used for univariate and multivariate analyses of variables related to OS.

**Conclusion:**

A significant association was found between the HB/RDW ratio and clinical characteristics and survival outcomes in ESCC patients. Based on these findings, we believe that the HB/RDW ratio is a novel and promising prognostic parameter for ESCC patients.

## INTRODUCTION

Esophageal cancer is one of the most common digestive malignancies worldwide [1, 2], leading to more than 20000 deaths every year in China [3, 4]. Esophageal squamous cell carcinoma (ESCC) is the predominant histological subtype of esophageal cancer in Asia [2, 3]. The clinical outcome of ESCC is poor, with the 5-year overall survival rate being less than 50% [5, 6]. At present, the prognosis of ESCC is commonly based on the tumor stage determined according to the TNM staging system [5, 6]. In addition, several prognostic factors associated with ESCC have been identified [7–9]. Baseline nutrition status and inflammation-based prognostic indicators, such as body mass index (BMI), the C-reactive protein/albumin ratio, the prognostic nutritional index (PNI) have been revealed as prognostic factors in ESCC [8, 10, 11]. Besides, several molecular and genetic biomarkers, such as the microRNA-3651, the overexpression and amplification of epithelial growth factor receptor (EGFR), SATB2 expression, has also showed promising prognostic value in ESCC by recent studies [12–14]. However, unconventional laboratory instruments and additional costs limit the application of these prognostic factors in the routine clinical practice. It is important to investigate and develop prognostic tools for ESCC from the perspective of clinical application as well as translational research.

Complete blood count (CBC) is a routine examination performed in cancer patients. Recently, the hematological parameters included in CBC have been shown to have prognostic significance in several cancers, for example, the hemoglobin (HB) level, combination of platelet count and mean platelet volume (COP-MPV) [15], platelet-to-lymphocyte ratio (PLR) and neutrophil-to-lymphocyte ratio (NLR) [16–20]. Low HB levels could reflect malnutrition in the host and the host immune status to some extent, both of which may indicate low tolerance to treatment. Several studies have demonstrated that anemia before treatment was a predictor of poor outcome in cancer patients, including patients with nasopharyngeal carcinoma, head and neck cancer, cervical cancer, ESCC and gastrointestinal cancer [16–19, 21, 22].

Another important CBC parameter in cancer is red cell distribution width (RDW), which is used to measure variability in the size of circulating red blood cells. Individual RDW values have been shown to be closely associated with poor outcome in cardiovascular diseases, pulmonary diseases and hepatic diseases [23–26]. More recently, several studies have explored its correlation with the clinical characteristics and prognosis of malignant diseases [19, 27–31]. High RDW values were found to correlate with advanced tumor stage and invasiveness in non-small-cell lung cancer [30, 32], breast cancer [28, 29, 31, 32], and renal cancer patients [29].

At present, consolidated evidence in the literature demonstrates that systematic inflammatory response is closely related with the development and progression of cancer, including ESCC [7, 8, 28, 33]. Several researchers have focused on the relationship between inflammatory status in the host and RDW and revealed a close correlation between the two [34, 35], and therefore, a high RDW value is associated with poor prognosis and aggressive behavior of cancer.

As mentioned above, previous studies have indicated that both HB and RDW are valuable prognostic factors in ESCC patients. Here, we proposed the use of a novel prognostic marker for patients with ESCC—the HB/RDW ratio, which was merging information from HB with RDW and was feasibly operated without additional costs. No study so far has assessed the clinical significance of the HB/RDW ratio in other cancers as well as ESCC, which makes this study the first of its kind. We aimed to evaluate the prognostic role of the HB/RDW ratio in a consecutive cohort of Chinese patients with locoregional ESCC, and to further explore the potential relationship between the HB/RDW ratio and the clinical characteristics of ESCC.

## RESULTS

### Patient characteristics and treatments

The data for 362 ESCC patients were analyzed (268 men and 94 women). The median age at the time of presentation with cancer was 58 years (mean, 57.96 years). Tumors were pathologically confirmed to be of a high or moderate differentiation grade in 270 patients (74.6%). Two hundred and twenty-nine tumors (63.3%) were limited to the middle third of the esophagus. Two hundred and three (56.1%) patients were categorized as having AJCC/UICC stage I/II disease. The Glasgow prognostic score (GPS) [36, 37] was calculated to evaluate the systemic inflammatory status of each patient. The baseline GPS was 0, 1, and 2 in 280 (77.3%), 69 (19.1%), and 13 (3.6%) patients, respectively. The NLR ranged between 0.63 and 59, and the mean and median of NLR were 2.9 and 2.2, respectively. The PLR ranged between 29 and 577, and the mean and median of PLR were 130 and 117, respectively. The study population presented with a median HB of 13.7 g/dL (range, 5.05–16.8 g/dL). 35 patients (9.7%) were evaluated as anemia. At initial diagnosis, the median RDW was 12.4%, and the mean RDW was 12.6%. The HB/RDW ratio was then calculated by dividing the HB value (g/dL) by the RDW value (%). The median and mean values of this ratio were 1.1059 and 1.0825, respectively. All the patients (*n* = 362) underwent radical esophagectomy. Two hundred and eighty-nine patients (79.8%) underwent radical esophagectomy alone and 73 (20.2%) underwent radical esophagectomy combined with adjuvant treatment.

### Survival analysis

**Figure 1:**
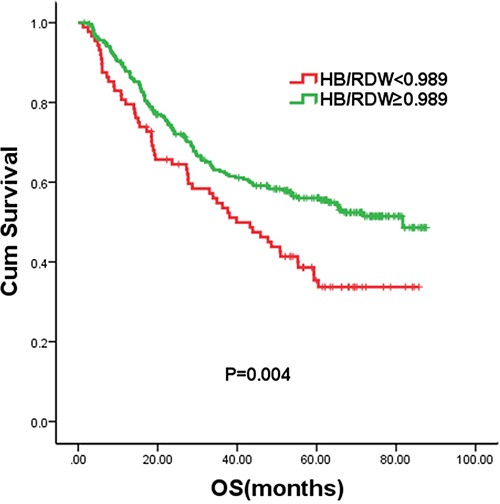
Kaplan–Meier curves for overall survival (OS) according to the HB/RDW ratio

The median follow-up time was 43.8 months (range, 1.2–87.6 months). One hundred and seventy-eight patients died from ESCC-related causes before the end of the follow-up period. Median overall survival time for the entire patient group was 63 months. The 3- and 5-year OS rates were 60.7% and 51%, respectively. The optimal cut-off value of the HB/RDW ratio was determined to be 0.989 for OS. According to the HB/RDW ratio, the patients were classified into the high HB/RDW (≥0.989) and low HB/RDW (<0.989) groups. This binary classification of the HB/RDW levels was applied in subsequent analyses. There were 88 ESCC patients with a low HB/RDW ratio and 274 patients with a high HB/RDW ratio. The 5-year OS of the low HB/RDW group and the high HB/RDW group were 33.7% and 55.5%, with the median OS time of 39.8 months and 81.7 months, respectively (*P*=0.004, Figure 1).

Compared with patients with a high HB/RDW ratio (≥0.989), the crude HR for those with a low HB/RDW ratio (<0.989) was 1.589 (*P* = 0.004). Univariate analysis indicated that lymph node status, tumor depth, treatment, GPS, tumor size and HB/RDW were significant predictors of the clinical outcome of ESCC. On multivariate analysis, HB/RDW, tumor depth and lymph node status were proved to be independent predictors of OS. GPS (*P* = 0.223) and tumor size (*P* = 0.982) were not significantly associated with OS after adjusting for other covariates. After adjusting for lymph node status, tumor depth, treatment, tumor size and GPS, we found that patients with a low HB/RDW ratio had a 1.416 times greater risk of dying during follow-up compared with those with a high HB/RDW (95% CI = 1.024–1.958, *P* = 0.035, Table 2). The stability of this model was therefore confirmed in a bootstrap resampling procedure. Among 1000 new models, the HB/RDW ratio remained to be an independent prognostic factor after adjustment (*P*=0.040).

**Table 2:** Univariate and multivariate analysis of OS in 362 ESCC patients

Variable	Univariate				Multivariate			
	P value	HR	95% CI		P value	HR	95% CI	
			Lower	Upper			Lower	Upper
**Gender**								
Male	Reference							
Female	0.396	0.863	0.614	1.213				
**Age(years)**								
≤60	Reference							
>60	0.875	0.976	0.724	1.317				
**Tumor grade**								
I-II	Reference							
III	0.241	1.216	0.877	1.687				
**Lymph nodes metastasis**								
Negative	Reference				Reference			
Positive	<0.001*	2.596	1.918	3.516	<0.001*	2.219	1.612	3.055
**Depth of tumor invasion**								
Tis-T2	Reference				Reference			
T3-T4	<0.001*	2.413	1.611	3.614	0.020*	1.677	1.084	2.596
**Tumor location**								
Upper	Reference							
Middle	0.898	0.968	0.590	1.589				
Lower	0.550	0.844	0.484	1.472				
**Treatment**								
Surgery alone	Reference				Reference			
Surgery+ adjuvant RT/CT	0.008*	1.577	1.127	2.207	0.665	1.081	0.759	1.540
**GPS**								
0	Reference				Reference			
1-2	0.033*	1.433	1.029	1.995	0.230	1.235	0.875	1.744
**Smoking**								
Never	Reference							
Ever	0.341	1.164	0.852	1.590				
**Tumor size (cm)**								
≤3.5	Reference				Reference			
>3.5	0.044*	1.353	1.008	1.817	0.982	1.004	0.736	1.369
**NLR**								
≤3	Reference							
>3	0.693	0.932	0.657	1.321				
**PLR**								
≤150	Reference							
>150	0.817	0.960	0.681	1.354				
**Hemoglobin (g/dL)**								
<10.3	Reference							
≥10.3	0.067	0.493	0.231	1.051				
**RDW (%)**								
<13.6	Reference							
≥13.6	0.094	1.381	0.946	2.016				
**HB/RDW**								
≥0.989	Reference				Reference			
<0.989	0.004*	1.589	1.156	2.185	0.035*	1.416	1.024	1.958

Abbreviations: OS=overall survival; ESCC=esophageal squamous cell carcinoma; HB=hemoglobin; RDW=red cell distribution width; GPS=Glasgow prognostic score; NLR=neutrophil to lymphocyte ratio; PLR= platelet to lymphocyte ratio; HR=hazard ratio; CI=confidence interval; RT=radiotherapy; CT=chemotherapy.

* *p* < 0.05

In addition, we replaced the HB/RDW with either HB or RDW and performed multivariate analyses of OS by Cox model. After adjusting for lymph node status, tumor depth, treatment, tumor size and GPS, neither HB nor RDW was found independently associated with OS (*P*=0.150, *P*=0.084, respectively). The Harrell's C-index for the HB/RDW, HB and RDW was 0.57, 0.45 and 0.35, respectively.

### Relationship between the HB/RDW ratio and clinicopathological features

**Figure 2:**
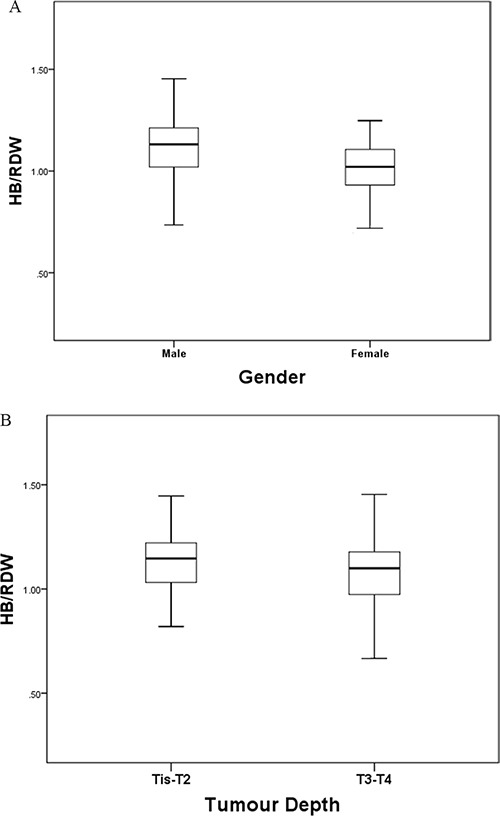
The HB/RDW ratio according to tumor depth A. and gender B. The HB/RDW ratio was significantly higher in male patients and patients with Tis/T1/T2 ESCC

We also explored the association between the HB/RDW ratio and other clinicopathological characteristics in ESCC patients. A significantly higher percentage of patients in the low HB/RDW group had T3/T4 ESCC (*P*=0.026), UICC/AJCC stage III disease (*P*=0.013), and underwent adjuvant therapy (*P*=0.022) (Table 1). The median HB/RDW ratio of male patients and female patients was 1.11 and 1.00 respectively (*P* < 0.001) (Figure 2A). ESCC patients with T3/T4 disease and those with UICC/AJCC stage III disease presented with a median HB/RDW ratio of 1.068 and 1.060, respectively (Figure 2B). Patients in the low HB/RDW group were found to present with higher NLR (*P*=0.004), PLR (*P*=0.001), RDW (*P*<0.001) and lower HB level (*P*<0.001) (Table 1). Meanwhile, it was found that patients with anemia had a significantly higher RDW compared with non-anemia group (13.3 vs. 12.5, *P*<0.001).

**Table 1:** Baseline clinical features of 362 patients with ESCC

Group	Total	%	HB/RDW				*p* value
			<0.989	%	≥0.989	%	
**Gender**							
Male	268	74.0	54	61.4	214	78.1	0.003*
Female	94	26.0	34	38.6	60	21.9	
**Age(years)**							
≤60	217	59.9	51	58.0	166	60.6	0.708
>60	145	40.1	37	42.0	108	39.4	
**Tumor grade**							
I-II	270	74.6	69	78.4	201	73.4	0.339
III	92	25.4	19	21.6	73	26.6	
**Lymph nodes metastasis**							
Negative	194	53.6	40	45.5	154	56.2	0.086
Positive	168	46.4	48	54.5	120	43.8	
**Depth of tumor invasion**							
Tis-T2	95	26.2	15	17.0	80	29.2	0.026*
T3-T4	267	73.8	73	83.0	194	70.8	
**TNM stage**							
I-II	203	56.1	39	44.3	164	59.9	0.013*
III	159	43.9	49	55.7	110	40.1	
**Tumor location**							
Upper	38	10.5	4	4.5	34	12.4	0.052
Middle	229	63.3	55	62.5	174	63.5	
Lower	95	26.2	29	33.0	66	24.1	
**GPS**							
0	280	77.3	65	73.9	215	78.5	0.382
1-2	82	22.7	23	26.1	59	21.5	
**Smoking**							
Never	129	35.6	38	43.2	91	33.2	0.097
Ever	233	64.4	50	56.8	183	66.8	
**Tumor size (cm)**							
≤3.5	186	51.4	37	42	149	54.4	0.050
>3.5	176	48.6	51	58	125	45.6	
**NLR**							
Median	2.2		2.1		2.2		0.004*
Mean±SD	2.9±4.0		3.9±6.9		2.5±2.3		
**PLR**							
Median	117		126		113		0.001*
Mean±SD	130±67		150±79		124±62		
**Hemoglobin(g/dL)**							
Median	13.7		11.7		14.1		<0.001*
Mean±SD	13.5±1.6		11.6±1.5		14.1±1.1		
**RDW (%)**							
Median	12.4		13.7		12.2		<0.001*
Mean±SD	12.6±1.1		13.7±1.5		12.2±0.7		
**Treatment**							
Surgery alone	289	79.8	63	71.6	226	82.5	0.022*
Surgery+ adjuvant RT/CT	73	20.2	25	28.4	48	17.5	

Abbreviations: ESCC=esophageal squamous cell carcinoma; HB=hemoglobin; RDW=red cell distribution width; GPS=Glasgow prognostic score; NLR=neutrophil to lymphocyte ratio; PLR= platelet to lymphocyte ratio; SD=standard deviation; RT=radiotherapy; CT=chemotherapy

* *p* < 0.05

## DISCUSSION

The role of RDW has been increasingly appreciated, as RDW has been shown to closely correlate with risk of cardiovascular diseases and systematic inflammatory status [34, 35]. Previous studies have identified RDW as an accurate predictor of inflammatory status of hepatitis B-infected patients, mortality of acute pancreatitis, and activity of inflammatory bowel disease [24, 26, 35]. Moreover, elevated RDW were found to be an indicator of risk and progression of multiple malignancies, while the prognostic value of RDW has also been discussed [28, 29, 31, 32]. Warwick et al [30] analyzed a cohort of patients with non small cell lung cancer and identified a robust association of RDW with long term survival. A retrospective study of symptomatic multiple myeloma by Lee et al [31] revealed elevated RDW as a predictor of enhanced systematic inflammation and poor survival. However, the data on RDW in ESCC was limited. As far as we know, there was only one study exploring the clinical significance of RDW in ESCC. A retrospective study by Chen et al on 277 Chinese patients revealed RDW to be a potential prognostic factor and established a nomogram that could accurately predict survival of ESCC patients based on the RDW values [38].

Because RDW is influenced by complex clinical conditions, the impact of RDW on ESCC is not only related to its correlation with the inflammatory response, but is also related to the overall sub-optimal health status, which indicates a decreased capacity for systemic repair, recovery, and oxygenation. Although previous studies have shown encouraging results, we considered that RDW itself without other indicators might not reflect the systematic inflammatory status and give the exact prognostic information. Previous studies have combined multiple inflammatory indicators to successfully establish a series of indices for cancer prognosis, such as GPS, PNI, CRP/albumin and et al. Moreover, few studies have already combined RDW and platelet count and explored the clinical significance of the RDW/platelet ratio. Cetinkaya et al [39] retrospectively analyzed a cohort of acute pancreatitis cases and identified the RDW/platelet ratio as a predictor of mortality. The prognostic value of the RDW/platelet ratio in myocardial infarction was also discussed [40, 41]. As HB is a well-established nutritional indicator and a potential prognostic factor in ESCC [16–19, 21, 22], we combined HB and RDW and built a novel prognostic index—the HB/RDW ratio.

In the current study, we firstly investigated the prognostic significance of HB/RDW in assessing the outcomes in ESCC patients. Our results demonstrated that a low HB/RDW ratio (<0.989) is significantly associated with poor clinical outcome and a 1.416-fold greater risk of death in ESCC patients, which was further validated by bootstrap resembling model. Neither in other cancer nor in ESCC had the significance of HB/RDW been investigated before. To the best of our knowledge, this is the first time that the HB/RDW ratio has been found to be a predictor of overall survival (OS) in patients with ESCC.

Compared with other prognostic indices, the HB/RDW ratio has several advantages in ESCC patients. As we know, both HB and RDW are influenced by various non-cancer-related conditions, and HB/RDW could therefore minimize the potential basis and theoretically reflect generalized health information, such as the nutrition status, inflammatory status, and immune function. Thus, the prognostic value of the HB/RDW ratio for ESCC patients would be more reliable than the effect of either HB or RDW, which was supported by our data. In the current study, we found that neither HB nor RDW was significantly correlated with survival outcome, while the Harrell's C-index for the HB/RDW was significantly higher than that of HB or RDW.

In order to eliminate any potential bias, we used the well-known inflammatory parameter GPS in the Cox regression model, while multivariate analysis showed that the HB/RDW ratio, but not GPS, was an independent prognostic factor. Similarly, NLR and PLR failed to display as prognostic factors in the current study. From this point of view, the HB/RDW ratio may have additional prognostic value over the GPS with regard to predicting OS in ESCC patients, and may be a significant coadjutant for other inflammation-related markers. Moreover, the HB/RDW ratio is easy to obtain from CBC and does not need expensive instruments, complex calculations and additional costs. Therefore, the implementation of the HB/RDW ratio is feasible and convenient in clinical practice, and may provide new insight into the interpretation of RDW data.

A low HB/RDW ratio was also found to be significantly associated with advanced cancer stage in the current study. Interestingly, the association between lymph nodes status and HB/RDW was identified with a marginal statistical significance (*P*=0.086) in our study, which was partly due to sample size. Forty-nine ESCC patients with an HB/RDW ratio of <0.989 (55.7%) and 110 ESCC patients with an HB/RDW ratio of ≥0.989 (40.1%) were classified as stage III disease, respectively (*P* = 0.013). Apparently, a low HB/RDW ratio was indicative of aggressive tumor behavior and advanced tumor stage. This particular finding was reasonable based on previous findings that both low HB levels and high RDW values are closely related to aggressive tumor behavior [26, 42–44]. We supposed that this ratio was therefore useful as a predictor of tumor aggressiveness and a tool for the differential diagnosis of ESCC. Since CBC is a routine test conducted in general health examination and follow-up, it would be easy to calculate the HB/RDW ratio and to explore its clinical significance in cancer prevention and cancer monitoring. In the case of ESCC patients who present with a gradually increasing HB/RDW ratio after undergoing curative treatment, intensive adjuvant therapy should be administered and the possibility of recurrence should be considered.

In the current study, there were some limitations that need to be acknowledged. One of the main limitations is the retrospective nature of this study. Unfortunately, the correlation between the HB/RDW and post-operative morbidity, such as pneumonia, was not investigated for incomplete data. Further, it is possible that the HB/RDW ratio was inevitably influenced by systematic inflammatory diseases, as it was impossible to exclude any potential inflammatory conditions. Finally, we did not use an external population to validate the prognostic value of the HB/RDW ratio. Therefore, future prospective studies are required to overcome these limitations.

In summary, this study has shown a significant association between the HB/RDW ratio and the clinical characteristics and survival outcomes in ESCC patients, which was not affected by adjustment for other risk factors. Thus, we believe that the HB/RDW ratio has potential as an inexpensive, convenient and feasible prognostic parameter for ESCC patients, and we suggest that it should be included to better predict prognosis and facilitate the management of these patients. Future studies exploring the clinical significance of HB/RDW in other cancers are also warranted.

## MATERIALS AND METHODS

### Ethics statement

All patients provided authorized and written informed consent for their data to be stored in the Sun Yat-Sen University Cancer Center database and to be used for research. Study approval was obtained from an independent ethics committee at the Cancer Center of Sun Yat-Sen University. This study was undertaken in accordance with the ethical standards of the World Medical Association's Declaration of Helsinki.

### Patients

Between January 2007 and December 2008, 362 consecutive patients with ESCC who visited Sun Yat-Sen University Cancer Center were retrospectively analyzed. All the cases included in the present study met the following criteria: (a) pathological diagnosis of ESCC in a localized or loco-regional stage (stages I–III according to the sixth edition of the AJCC/UICC TNM system), (b) availability of complete clinical data and disease records, and (c) treatment with radical esophagectomy. The exclusion criteria were as follows: (a) with clinical evidence of infection or other inflammatory disease, (b) underwent preoperative treatment and (c) previously diagnosed as anemia.

Basic demographic characteristics (gender and age), detailed medical history and medications, and baseline tumor characteristics (grade and stage) of all the patients were collected (Table 1). Smoker was defined as ≥1 lifetime pack-years. Inflammatory indices including NLR, PLR and GPS were calculated and analyzed as reported by previous studies [45–48]. CBC was obtained within 2 weeks before the surgical procedure. On cessation of treatment, each patient was followed up every 3 months with an interview conducted at the clinic or over the telephone; each patient was followed up for at least 5 years. The last follow-up was on July 31, 2014.

### Statistical analyses

Differences in the relationship between the HB/RDW ratio and categorical clinicopathological features were assessed using the chi-square test and T test. We have introduced the method established by Jan Budczies et al [49] (at http://molpath.charite.de/cutoff/) to determine the optimal cutoff values for the HB/RDW ratio, the HB and the RDW. The overall survival (OS) was considered as the period from the date of diagnosis to the date of death or the last follow-up. In the case of patients who were alive, data obtained on the date of the last contact were censored. The Kaplan-Meier method was used to estimate the 5-year OS, while the log-rank test was used to determine differences in survival. The Cox proportional hazards model was used to determine the hazard ratio (HR) of variables related to OS and DFS in univariate and multivariate analyses. Bootstraps with 1000 resample were used to test the stability of the Cox model. Cox regressions with the same conditions as in the original data set were then calculated for the new data sets in order to obtain the bootstrap parameter estimates. The predictive accuracy of prognostic factor was evaluated by Harrell's concordance index (C-index) by R version 3.2.4 (http://www.r-project.org/). HRs with 95% confidence intervals (CIs) and two-sided p values were reported. *P* values <0.05 were considered to indicate statistical significance. All statistical analyses were performed using the Statistical Package for the Social Sciences (SPSS version 19.0, USA) and R version 3.2.4.
